# Exploring practitioners’ perceptions of health behavior changes associated with psychedelic experiences

**DOI:** 10.1038/s41598-025-25818-3

**Published:** 2025-11-25

**Authors:** Laura C. Carvalho, Jorge Encantado, Michiel van Elk, Arlen C. Moller, Talea Cornelius, Christopher Timmermann, Diogo Veiga, Pedro J. Teixeira

**Affiliations:** 1 Centro Interdisciplinar para o Estudo da Performance Humana (CIPER), Faculty of Human Kinetics, University of Lisbon, Lisbon, Portugal; 2 Cognitive Psychology Unit, Leiden University, Leiden, Netherlands; 3 Department of Psychology, Illinois Institute of Technology, Chicago, USA; 4 Center for Behavioral Cardiovascular Health, Columbia University Irving Medical Center, New York, US; 5 Department of Experimental Psychology, UCL, London, UK; 6 DMT Research Group, Department of Brain Sciences, Imperial College London, London, UK

**Keywords:** Health behavior change, Psychedelics, Psychedelic practitioners, Diet, Physical activity, Lifestyle modification, Preventive medicine

## Abstract

In addition to evidence supporting the use of psychedelics in treating alcohol and tobacco misuse, emerging research suggests that psychedelic experiences may also facilitate beneficial changes in other health behaviors, such as diet, exercise, and meditation. This retrospective, survey-based study explored perceived health behavior changes following psychedelic experiences, from the perspective of 96 psychedelic practitioners, working in both legal and underground contexts. Practitioners reported on perceived changes in their clients’ or patients’ behaviors, including physical activity, diet and nutrition, contemplative practices, and time spent in nature, as well as on their own behavior changes, and potential mechanisms underlying these changes. Behaviors with the highest proportion of clients perceived as having changed in a positive direction included contemplative practices (61.4%), time spent in nature (60.1%), eating patterns (56.9%), and diet and nutrition (56%). These findings suggest that practitioners often perceive psychedelic experiences as associated with positive health behavior change. Future research is needed to validate these findings, explore the extent and mechanisms of potential changes, and assess whether such effects are sustained over the long-term. Understanding these effects may help inform future behavior change interventions and public health policy.

**Supplementary Information:**

The online version contains supplementary material available at 10.1038/s41598-025-25818-3.

## Introduction

Lifestyle behaviors, such as engaging in regular physical activity, maintaining a healthy diet, refraining from tobacco use and alcohol consumption, and getting high quality sleep are known to be critical determinants of health and well-being^1^. Failure to engage in these healthy lifestyle behaviors is consistently and robustly associated with poor outcomes such as elevated risk of overweight or obesity, greater incidence of cardiovascular disease, diabetes, cancer and other non-communicable diseases, and, ultimately, premature death^2^. In 2022, 59% of the global adult population was living with excess weight or obesity^3^. Along with diabetes and other non-communicable diseases, these conditions are often caused by a combination of low levels of physical activity, high levels of sedentary behavior, and an imbalanced, high caloric diet^2^. Additionally, according to data from the World Health Organization (WHO), tobacco use contributes to 7 million deaths annually^4^, while insufficient physical activity increases by 20% to 30% the risk of death^5^.

 Improving healthy lifestyle behaviors is a promising target for interventions to reduce morbidity and mortality. A wide range of interventions have been developed to improve healthy lifestyle behaviors, including individual-level strategies such as behavioral counseling, motivational interviewing, and cognitive-behavioral approaches, as well as digital and mobile health interventions and structured programs targeting physical activity, diet, and smoking cessation^6^. Despite being grounded in rigorous scientific research and well-established theoretical frameworks^7^, these interventions have not been sufficiently effective in producing long-term behavioral change^8^. Additionally, rates of deaths related to non-communicable diseases remain high among the adult population^1^.

Several previous studies suggest that the use of psychedelics - psychoactive substances that can induce altered states of consciousness – in both controlled clinical settings and informal contexts, may be positively associated with healthy lifestyle and health behavior changes (see Teixeira and colleagues for a review^9^ [note: in this study, when we refer to ‘psychedelics’, we use an inclusive definition, referring to the ‘classic’ psychedelics, which include LSD, psilocybin mushrooms/truffles, DMT, ayahuasca and mescaline (natural or synthetic), as well as ketamine, MDMA and ibogaine, often considered “atypical” psychedelics]. To date, the most extensively studied health behaviors, in relation to psychedelics, lie at the intersection of behavioral medicine and mental health, involving alcohol and tobacco (mis)use. In the first randomized double-blind trial of psilocybin-assisted psychotherapy for alcohol dependence, self-reported heavy drinking days, during a follow-up period of eight months, were significantly lower in the group that received two psilocybin sessions in adjunction to psychotherapy, when compared to active placebo and psychotherapy^10^. Preceding this study, a series of cross-sectional observational studies showed that the use of psychedelics was associated with decreased alcohol consumption^11–14^. For tobacco cessation or reduction there is also evidence from both clinical trials^15^ and observational studies^16–18^^19^.

The use of psychedelics may also be associated with protective benefits more broadly. In cross-sectional population-based studies, individuals reporting lifetime classic psychedelics use had lower odds of heart disease and diabetes in the past year^20^, lower odds of being overweight or obese, and higher odds of greater self-reported overall health^21^, compared to non-users. Another study found that individuals who used classic psychedelics reported healthier behaviors related to tobacco use and diet, with greater psychological insight during their most significant psychedelic experience linked to healthier exercise habits and a higher likelihood of maintaining a healthy body mass index^22^. In addition, data from two studies comparing public health indicators between ayahuasca users and the general population, in Spain and The Netherlands, showed that ayahuasca users reported healthier diet patterns, higher levels of physical activity, less use of medication, and improved well-being^23,24^.

Despite the halt in psychedelic research in the 1970 s and the imposition of legal restrictions^25^, psychedelic rituals and ceremonies continued to be practiced, both as part of indigenous traditions, and in underground settings in western countries, typically involving practitioners assisting and guiding these experiences (sometimes called “facilitators” or “guides”). Around the world, thousands of people take part in either legal or underground “psychedelic retreats” (and in a range of other settings), where a facilitator or guide plays a key role. Determining the exact number of individuals attending guided psychedelic retreats annually is challenging due to the lack of comprehensive data. However, interest in such retreats has grown significantly over the past decade. A study by the travel platform Booking found that 14% of travelers expressed interest in psychedelic retreats^26^ and, according to data published by the psychedelic retreat platform Retreat Guru^27^, the number of psychedelic retreat destinations worldwide has increased dramatically in the last decade. The site lists 1047 Ayahuasca and 680 psilocybin retreat destination options in March 2025. Additionally, revenue generated by its listed retreats increased from $1.6 million in the first quarter of 2019 to $5.1 million by the second quarter of 2023^28^. Data from the Global Psychedelic Survey^29^ revealed that among a worldwide sample of 5,108 English-speaking adults reporting lifetime psychedelic use, 17.8% accessed the substances through a shaman, while 13.4% obtained them via an underground therapist.

Additionally, with the growing body of research on psychedelic-assisted therapy for multiple mental health conditions (e.g., treatment-resistant depression), and the availability of ketamine-assisted therapy services, licensed health professionals are also providing support and guidance to individuals during psychedelic experiences. Nonetheless, licensed health professionals working in legally sanctioned settings represent a small minority of guided psychedelic experiences each year. For this reason, it may be important to also include underground practitioners in research, as excluding them risks overlooking valuable know-how derived from their practical experience and observations.

Extant psychedelic research predominantly focuses on the attitudes and knowledge of mental health professionals and specialists regarding their perspective around psychedelic-assisted psychotherapy, who mostly do not have practical experience in supporting others^30–36^. The few studies focusing on psychedelic practitioners have explored their perspective on preparation and integration practices^37,38^ and the therapeutic potential of psychedelics for eating disorders^39^. However, the unique perspectives of underground psychedelic practitioners may enrich our understanding of many other aspects surrounding psychedelic experiences, including the impact of psychedelics on a range of relevant outcomes, such as changes in health behaviors or lifestyle.

To our knowledge, this study is the first to examine psychedelic practitioners’ perceptions regarding the impact of psychedelic use on health-related behaviors. Given that these professionals may closely accompany and support individuals throughout the various stages of a psychedelic experience^40,41^ their insights offer valuable contributions to the broader understanding of these phenomena, complementing existing research on the topic.

## Purpose of the present study

The present study represents a novel approach to investigate the potential of psychedelics to influence health behaviors in users by acquiring perspectives from psychedelic practitioners who have had multiple experiences (and untapped knowledge) in this domain. Psychedelic practitioners are defined here as individuals who guide or assist others through their psychedelic experiences, in both legal (e.g., clinical trials, ketamine clinics) and underground contexts (e.g., retreats for individuals or groups). Psychedelic practitioners might be paid professionals or unpaid volunteers, and can be referred to as guides, facilitators, therapists, shamans, or various other titles. Our aim was to assess psychedelic practitioners’ perceptions of health behavior changes in clients or patients following one or more psychedelic experiences, and also their views on the psychosocial mechanisms that may be involved Understanding these mechanisms is crucial for optimizing intervention outcomes, by explaining how psychedelic experiences may lead to changes in health behaviors. In addition, both practitioners’ own self-reported changes in health-related behaviors, and the frequency with which clients or patients mentioned these behavior changes during their interactions with practitioners, were assessed.

## Results

### Demographic characteristics

A total of 103 psychedelic practitioners participated in the survey. Seven participants were later excluded for not meeting the inclusion criteria of having had at least ten personal psychedelic experiences and/or not having guided at least 30 psychedelic experiences. This resulted in a final sample of 96 participants.

Likely because of the private and sensitive nature of this practice under most legal jurisdictions, many participants (*n* = 73) chose not to provide personal information including biographical information, lifetime psychedelic use, and experience as practitioners (these participants were included in the analysis reported below). Almost half of respondents (mean age = 45 [SD = 9.41]) identified as female (47.8%), with 43.5% reporting ‘master’s degree’ as the highest level of education and 56.5% as being self-employed (see Tables 1 and 2).

**Table 1:** Sample characteristics (*n* = 23).

Age, mean (SD)	45 (9.41)
Female (%)	47.80%
Highest educational level (%)	
*Less than high school*	0%
*Graduated high school*	4.30%
*Trade/technical school*	4.30%
*Some college*,* no degree*	8.70%
*Bachelor’s degree*	8.70%
*Master’s degree*	43.50%
*Doctorate or professional degree*	30.40%
Employment status	
*Employed full-time (40 or more hours per week)*	30.40%
*Employed part-time (up to 39 h per week)*	4.30%
*Unemployed*	4.30%
*Student*	0%
*Retired*	4.30%
*Homemaker*	0%
*Self-employed*	56.50%
*Unable to work*	0%

**Table 2:** Lifetime experience as a psychedelic practitioner and lifetime psychedelic use (*n* = 23).

Number of experiences facilitated	
*Mean*	272.39
*SD*	439.24
*Median*	100
*Range*	30–2000
N; % substances worked with (most often worked with)	
*Psilocybin mushrooms*	19; 82.6% (5; 21.4%)
*Psilocybin truffles*	9; 39.1% (2; 8.7%)
*Synthetic psilocybin*	5; 21.7% (1; 4.3%)
*Ayahuasca*	13; 56.5% (7; 30.4%)
*DMT*	4; 17.4% (0%)
*Peyote/San Pedro*	8; 34.8% (1; 4.3%)
*LSD*	8; 34.8% (1; 4.3%)
*MDMA*	11; 47.8% (2; 8.7%)
*Ketamine*	9; 39.1% (3; 13%)
*Ibogaine*	1; 4.4% (0%)
*Combination substances*	9; 39.1% (1; 4.3%)
*Other*	2; 8.7% (0%)
Number of experiences had	
*Mean*	156.96
*SD*	184.54
*Median*	100
*Range*	10–800
N; % substances used (N; % most often used)	
*Psilocybin mushrooms*	19; 82.6% (6; 26.1%)
*Psilocybin truffles*	13; 56.5% (0%)
*Synthetic psilocybin*	4; 17.4% (0%)
*Ayahuasca*	21; 91.3% (8; 34.8%)
*DMT*	11; 47.8% (0%)
*Peyote/San Pedro*	19; 82.6% (2; 8.7%)
*LSD*	16; 69.6% (2; 8.7%)
*MDMA*	17; 73.9% (4; 17.4%)
*Ketamine*	14; 60.9% (0%)
*Ibogaine*	2; 8.7% (0%)
*Combination substances*	11; 47.8% (1; 4.3%)
*Other*	7; 30.4% (0%)

The number of psychedelic experiences facilitated by practitioners ranged from 30 to 2000 (M = 272.39; SD = 439.24; Md = 100) with an interquartile range of 150, from 50 (Q1) to 200 (Q3).

Participants reported a range of substances they used in their sessions, including psilocybin mushrooms (82.6%), ayahuasca (56.5%), MDMA (47.8%) and psilocybin truffles (39.1%). The substance most frequently reported as the “most frequently used” was ayahuasca (30.4%). Practitioners’ own lifetime use ranged from 10 to 800 experiences (M = 156.96; SD = 184.54; Md = 100) and ayahuasca was the most frequently used psychedelic (34.8%).

### Perceptions of change in clients/patients

**Fig. 1:**
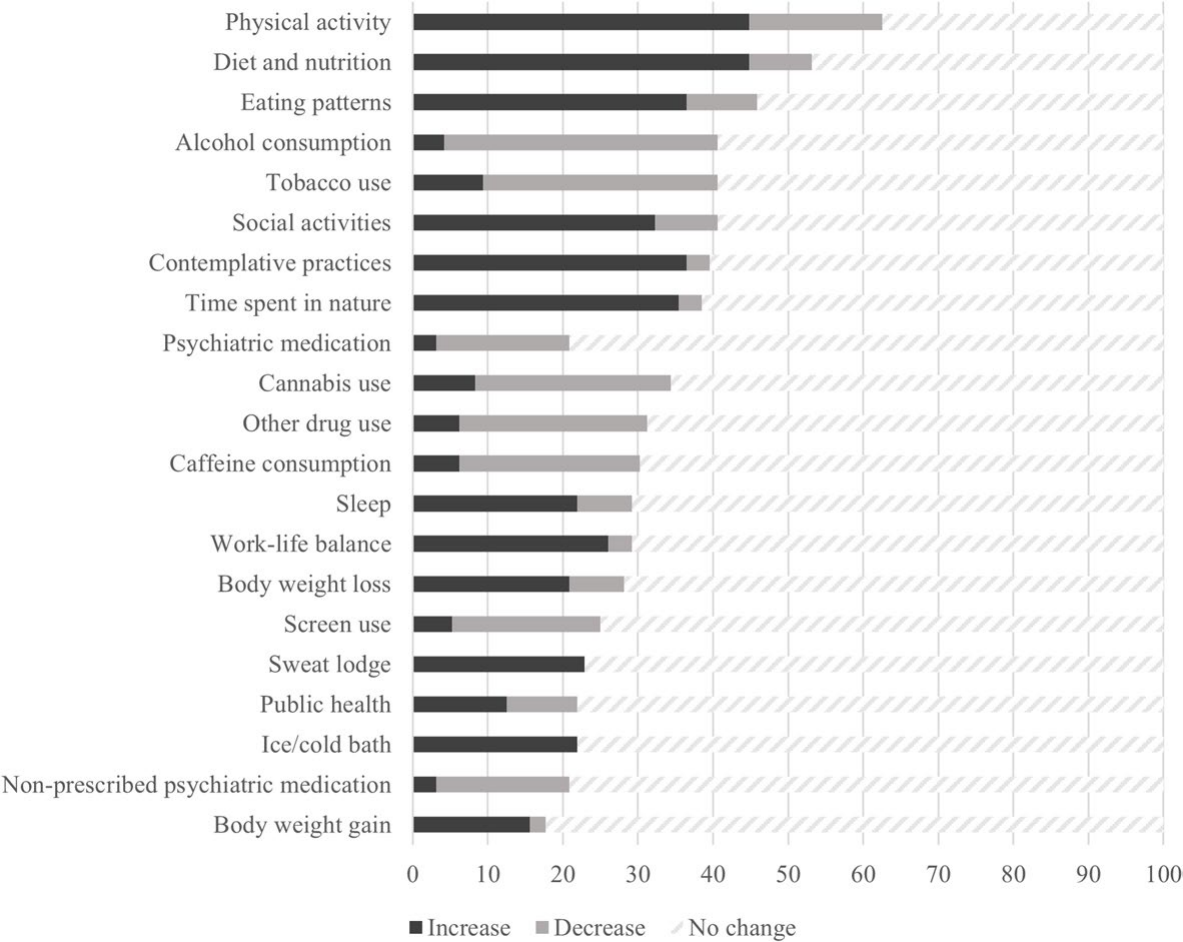
Percentage of practitioners reporting changes in clients’ or patients’ health-related behaviors (*n* = 96).

The behaviors with the majority of practitioners reporting perceived changes were physical activity (62.5%) and diet and nutrition (53.1%), followed by 40.6% of participants reporting changes in tobacco use, alcohol consumption and social activities. Around 30% of participants reported changes in contemplative practices (39.6%), time spent in nature (38.5%), psychiatric medication use (35.4%), other drug use (31.3%), and caffeine consumption (30.2%). More than 70% of practitioners reported no change in sleep (70.8%), weight loss (71.9%), screen use (75%), sweat lodge/sauna use (77.1%), ice/cold bath or shower (78.1%), public health recommendations (78.1%), non-prescribed psychiatric medication use (79.2%), and weight gain (82.3%). See Fig. 1 for detailed information on the percentage of practitioners reporting increases or decreases for each behavior.

Practitioners who reported perceiving changes were asked to estimate the proportion of their clients who showed increases versus decreases in each behavior **(T**able 3**).**

**Table 3:** Proportion of clients or patients with perceived increases or decreases in health-related behaviors

Behavior	% Practitioners reporting change	Perceived % of clients with increases (M ± SD)	Perceived % of clients with decreases (M ± SD)
Physical activity	62.5	46.7 ± 25.1	24 ± 27.8
Diet and nutrition	53.1	56 ± 23.5	25.1 ± 28.7
Eating patterns	45.8	56.9 ± 26.6	22.3 ± 29.1
Tobacco use	40.6	23.2 ± 23.8	47.2 ± 29.6
Alcohol consumption	40.6	42.3 ± 42.7	53.1 ± 26.2
Social activities	40.6	55.2 ± 25.6	26.6 ± 12.3
Contemplative practices	39.6	61.4 ± 24.2	6 ± 1
Time spent in nature	38.5	60.1 ± 27.6	17.7 ± 14.5
Psychiatric medication use	35.4	12.4 ± 8.9	53.4 ± 31.8
Cannabis use	31.3	35.4 ± 21.1	33 ± 21.4
Other drug use	31.3	17 ± 14.8	60.8 ± 27.7
Caffeine consumption	30.2	38.5 ± 35	44.3 ± 23.2
Sleep	29.2	50.3 ± 28	30.6 ± 16
Work-life balance	29.2	43.7 ± 26.1	29.3 ± 24
Weight loss	28.1	40.9 ± 28.4	18.4 ± 22.4
Screen use	25	21.8 ± 23.8	47.2 ± 25.4
Sweat lodge/sauna use	22.9	40 ± 29.7	0
Ice/cold bath or shower	21.9	32.7 ± 27.1	0
Public health recommendations	21.9	35.1 ± 24.2	36.4 ± 20.9
Non-prescribed psychiatric med use	20.8	33.7 ± 36.1	44.5 ± 34.3
Weight gain	17.7	40.2 ± 28.8	39.5 ± 51.6

The behaviors with the highest proportion of clients with perceived ***increases*** were contemplative practices (mean percentage = 61.4%; SD = 24.2), time spent in nature (mean = 60.1%; SD = 27.6), personally meaningful social activities (mean = 55.2%; SD = 25.6), and sleep (mean = 50.3%; SD = 28). Practitioners also reported mainly healthier eating patterns (mean = 56.9%; SD = 26.4), and diet and nutrition (mean = 56%; SD = 23.5). In addition, physical activity, cannabis use, work-life balance, weight loss, sweat lodge/sauna use, ice/cold bath or shower, and weight gain were also perceived more frequently as increasing rather than decreasing.

Conversely, the behaviors with the highest proportion of clients with perceived ***decreases*** were other drugs use (mean = 60.8%; SD = 27.7), alcohol consumption (mean = 53.1%; SD = 26.2), psychiatric medication use (mean = 53.4%; SD = 31.8), tobacco use (mean = 47.2%; SD = 29.6), and screen use (mean = 47.2%; SD = 25.4). Caffeine consumption, compliance with public health recommendations, and non-prescribed psychiatric medication use were also more frequently perceived as decreasing rather than increasing.

Regarding complex behaviors (e.g., physical activity), practitioners who perceived changes in these behaviors reported whether each specific activity or practice (e.g., walking) had increased or decreased (binary scale). Most practitioners (i.e., *n* = 28–60) perceived increases among their clients across all specific physical activities, contemplative practices, personally meaningful social activities, and work-life balance. Additionally, only increases were perceived for all specific time spent in nature practices.

Within diet and nutrition, increases were mostly perceived for the consumption of “vegan/vegetarian meals” (*n* = 33; 64.7%) and “legumes” (*n* = 22; 43.1%). For “vegetables and fruits” (*n* = 39; 76.5%%) and “nuts/whole grains” (*n* = 26; 51%), all participants reported perceived increases in consumption. Conversely, most practitioners perceived decreases in the consumption of “meat” (*n* = 28; 54.9%), while for “sugar-based foods and drinks” (*n* = 35; 68.6%) and “processed foods” (*n* = 31; 60.8%) all participants reported perceived decreases in consumption. Finally, for specific eating and relation with food patterns, mostly increases were perceived for “flexible eating” (*n* = 22; 50%) and “enjoyment of a broader range of foods without guilt” (*n* = 21; 47.7%). A majority of practitioners reported perceived decreases in “binges or cravings” (*n* = 23; 52.3%) and “purgative behaviors” (*n* = 15; 34.1%). Additionally, all participants reported perceived increases for the eating patterns “slow mindful eating” (*n* = 26; 59.1%), “eating according to one’s body needs” (*n* = 29; 65.9%), and “ability to balance calories in vs. calories out” (*n* = 16; 36.4%).

For detailed information see Supplementary File_1.

### Mentions by patients or clients before, during and after sessions Only twenty-four participants responded to the frequency of conversations around health-related behavior changes initiated by their clients before and after the psychedelic experience, and 23 responded for the period during the psychedelic experience.

*Before* the psychedelic experience, the three behavior changes that practitioners reported as most mentioned were work-life balance (*n* = 16), personally meaningful social activities (*n* = 13), and contemplative practices (*n* = 13).

In general, *during* the psychedelic experience, the majority of variables (e.g., body weight; *n* = 20) were rated with a frequency of “rarely” by most participants, with the exception of contemplative practices (*n* = 11), time spent in nature (*n* = 13) and personally meaningful social activities (*n* = 11), rated by the majority of participants with a frequency of “sometimes”, and work-life balance reported with an equal frequency of “sometimes” and “often/very often“ (n = 8).

Finally, in the period *after* the psychedelic experience (i.e., integration phase), six health behavior changes were reported as most frequently mentioned “often/very often”: work-life balance (*n* = 18), personally meaningful social activities (*n* = 18), time spent in nature (*n* = 17), physical activity (*n* = 14) and diet and nutrition (*n* = 12). For more detailed information, see Table 4.

**Table 4:** Health behavior mentions by patients or clients.

Behaviors	Rarely		Sometimes		Often/very often	
	*n*	%	*n*	%	*n*	%
Before the psychedelic experience (i.e., preparation) (*n* = 24)						
Physical activity	6	25%	10	41.7%	8	33.3%
Diet and nutrition	5	20.8%	9	37.5%	10	41.7%
Eating patterns	5	20.8%	9	37.5%	10	41.7%
Body weight	12	50%	8	33.3%	4	16.7%
Alcohol consumption	5	20.8%	7	29.2%	12	50%
Tobacco use	9	37.5%	8	33.3%	7	29.2%
Caffeine consumption	13	54.2%	7	29.2%	4	16.7%
Cannabis use	9	37.5%	7	29.2%	8	33.3%
Other drug use	11	45.8%	7	29.2%	6	25%
Non-prescribed med. use	10	41.7%	8	33.3%	6	25%
Psychiatric med. use	6	25%	8	33.3%	10	41.7%
Contemplative practices	4	16.7%	7	29.2%	13	54.2%
Time spent in nature	3	12.5%	10	41.7%	11	45.8%
Personally meaningful social activities	1	4.2%	10	41.7%	13	54.2%
Sleep	2	8.3%	11	45.8%	11	45.8%
Compliance with public health recommendations	14	58.3%	7	29.2%	3	12.5%
Screen use	9	37.5%	9	37.5%	6	25%
Work-life balance	2	8.3%	6	25%	16	66.7%
Ice/cold bath (or shower)	17	70.8%	4	16.7%	3	12.5%
Sweat lodge/sauna use	17	70.8%	6	25%	1	4.2%
During the psychedelic experience (*n* = 23)						
Physical activity	18	78.3%	2	8.7%	3	13%
Diet and nutrition	16	69.6%	3	13%	4	17.4%
Eating patterns	14	60.9%	6	26.1%	3	13%
Body weight	20	87%	2	8.7%	1	4.3%
Alcohol consumption	14	60.9%	6	26.1%	3	13%
Tobacco use	15	65.2%	6	26.1%	2	8.7%
Caffeine consumption	19	82.6%	1	4.3%	3	13%
Cannabis use	13	56.5%	9	39.1%	1	4.3%
Other drug use	16	69.6%	3	13%	4	17.4%
Non-prescribed med. use	16	69.6%	4	17.4%	3	13%
Psychiatric med. use	15	65.2%	3	13%	5	21.7%
Contemplative practices	6	26.1%	11	47.8%	6	26.1%
Time spent in nature	4	17.4%	13	56.5%	6	26.1%
Personally meaningful social activities	6	26.1%	11	47.8%	6	26.1%
Sleep	13	56.5%	6	26.1%	4	17.4%
Compliance with public health recommendations	20	87%	2	8.7%	1	4.3%
Screen use	18	78.3%	3	13%	2	8.7%
Work-life balance	7	30.4%	8	34.8%	8	34.8%
Ice/cold bath (or shower)	20	87%	2	8.7%	1	4.3%
Sweat lodge/sauna use	20	87%	2	8.7%	1	4.3%
After the psychedelic experience (i.e.,integration) (*n* = 24)						
Physical activity	5	20.8%	5	20.8%	14	58.3%
Diet and nutrition	4	16.7%	8	33.3%	12	50%
Eating patterns	5	20.8%	9	37.5%	10	41.7%
Body weight	10	41.7%	10	41.7%	4	16.7%
Alcohol consumption	2	8.3%	12	50%	10	41.7%
Tobacco use	8	33.3%	12	50%	4	16.7%
Caffeine consumption	13	54.2%	7	29.2%	4	16.7%
Cannabis use	9	37.5%	9	37.5%	6	25%
Other drug use	12	50%	6	25%	6	25%
Non-prescribed med. use	13	54.2%	5	20.8%	6	25%
Psychiatric med. use	10	41.7%	5	20.8%	9	37.5%
Contemplative practices	2	8.3%	9	37.5%	13	54.2%
Time spent in nature	2	8.3%	5	20.8%	17	70.8%
Personally meaningful social activities	1	4.2%	5	20.8%	18	75%
Sleep	4	16.7%	10	41.7%	10	41.7%
Compliance with public health recommendations	16	66.7%	7	29.2%	1	4.2%
Screen use	10	41.7%	8	33.3%	6	25%
Work-life balance	3	12.5%	3	12.5%	18	75%
Ice/cold bath (or shower)	15	62.5%	8	33.3%	1	4.2%
Sweat lodge/sauna use	16	66.7%	6	25%	2	8.3%

### Psychosocial mechanisms for health behavior change

Only twenty-seven participants responded about the possible mechanisms involved in the association between changes in health-related behaviors and psychedelic experiences. Four participants were removed from the sample due to incorrect answers to the “attention check” question, resulting in a final sample of 23 participants.

**Fig. 2:**
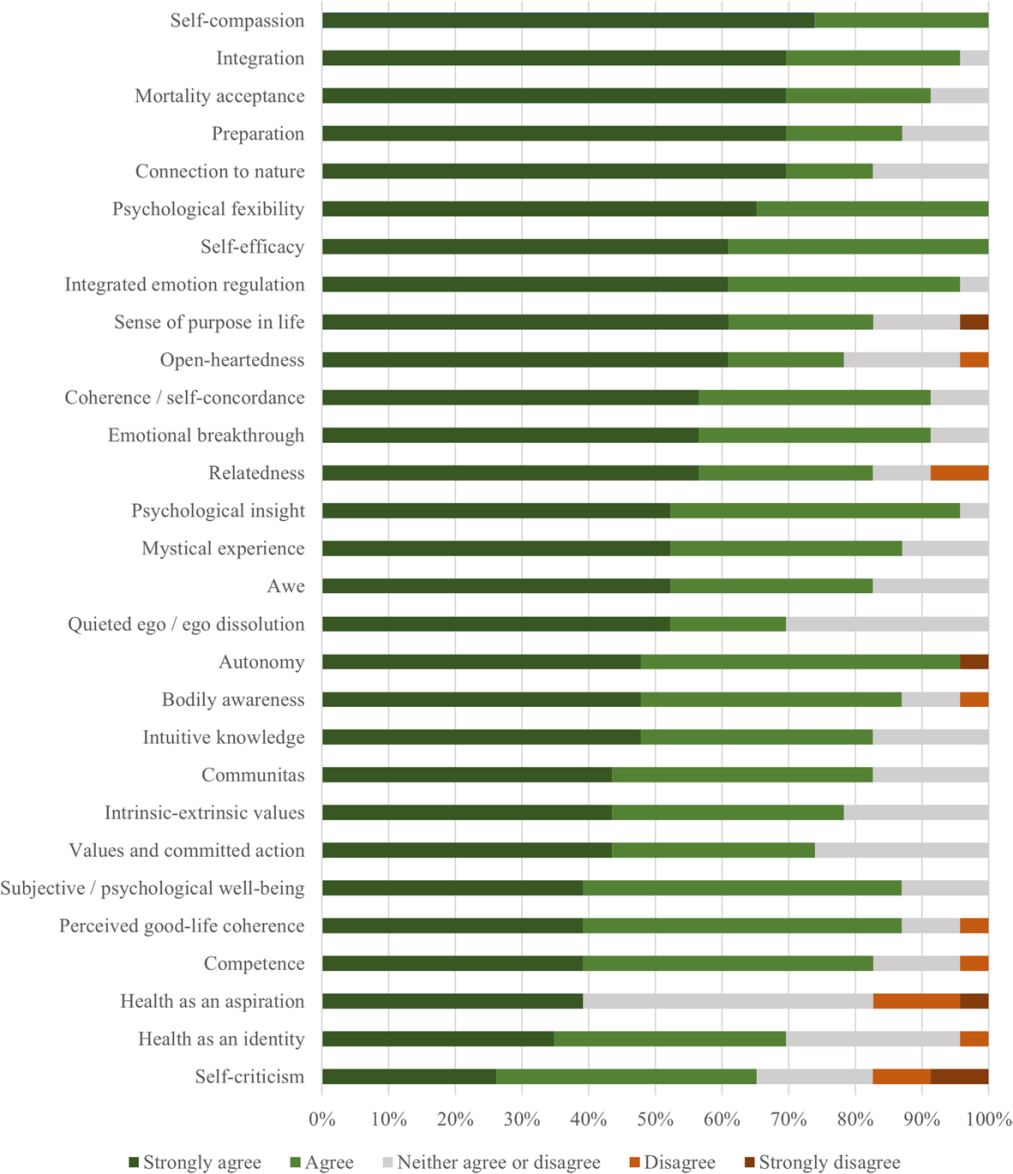
Association between psychological mechanisms and health behavior change (*n* = 23).

The means (from a five-point scale from strongly disagree to strongly agree) were consistently high and closely aligned across all concepts presented, with the most common response for the majority (17/29; e.g., self-efficacy) of the psychological mechanisms being “strongly agree”. The ten psychological mechanisms with the highest mean scores were self-compassion (M = 4.74), psychological flexibility (M = 4.65), self-efficacy (M = 4.61), mortality acceptance (M = 4.61), integrated emotion regulation (M = 4.57), connection with nature (M = 4.52), coherence/self-concordance (M = 4.48), emotional breakthrough (M = 4.48), and psychological insight (M = 4.48). Opposingly, the five psychological mechanisms with the lowest mean scores were competence (M = 4.17), health as an aspiration (M = 4.17), values and committed action (M = 4.17), health as an identity (M = 4.00), and decreased self-criticism (M = 3.65). Although not strictly a psychological mechanism, preparation and integration practices were also scored highly by participants. For more detailed information, see Fig.2.

### Practitioners’ own health behavior change

In addition to reporting perceived behavior changes in their clients, a small subset of participants (*n* = 27) also reported changes in their own health behaviors as a result of their psychedelic experiences. The majority reported changes in contemplative practices (*n* = 27), time spent in nature (*n* = 26), eating patterns and relationship with food (*n* = 25), personally meaningful social activities (*n* = 24), diet and nutrition (*n* = 26), physical activity (*n* = 24), and work-life balance (*n* = 19). The changes reported were mostly in a healthier direction, with practitioners engaging in more contemplative practices and physical and social activities, as well as having a healthier diet and nutrition/eating patterns, and enhanced work-life balance.

For detailed information on psychedelic practitioners’ self-reported health behavior changes, see Supplementary File_2.

## Discussion

Psychedelic practitioners, whether operating underground or in legal contexts, have a unique vantage point on their clients’ or patients’ psychedelic experiences and may also have the opportunity to witness the transformations that may follow. The main goal of this study was to explore practitioners’ perceptions of health behavior change and the mechanisms by which they believe these changes occur, offering an indirect avenue for understanding the role psychedelics may play in motivating health behavior changes. This form of observer data from the practitioners represents a methodological complement to first-person accounts of (one’s) behavior, which have to-date dominated the empirical literature exploring these questions^e.g.,22^.

Data from the current study suggest that, with the exception of physical activity and diet and nutrition, the majority of practitioners perceive behaviors to be unchanged. However, among the minority of practitioners who did report perceived changes, these were almost invariably described as positive, encompassing increases in health-promoting behaviors such as physical activity and improved diet and nutrition patterns, along with decreases in health-hindering behaviors such as alcohol and tobacco use. This health-promoting pattern included several other behaviors less commonly investigated in behavioral health research, such as increases in contemplative practices, time spent in nature, personally meaningful social activities, and a reduction in other non-psychedelic substance use. Among the small sample of practitioners who reported their own behavior changes, the same behaviors were most frequently identified as having positively changed.

The perceptions of psychedelic practitioners are consistent with evidence from studies investigating psychedelic users, showing that psychedelics are perceived by some individuals to assist or somehow influence individuals towards healthier living^9^. In one of the earliest studies of psychedelic-assisted therapy for depression^42^, when participants were asked about other changes in their life, nearly half of the sample reported *“improvements to diet*,* exercise*,* and cutting down on drinking alcohol”* (p. 532). Participants in subsequent clinical trials^18,43^ and observational studies^44,45^ also reported positive changes in health-related behaviors, such as diet, physical activity and tobacco use. It is worth noting that these reported changes were likely spontaneous, meaning that the psychedelic users most likely did not intend to change specific health-behavior by having a psychedelic experience (except if that was the focus of the study, i.e., nicotine addiction).

 Although motives for participation were not specifically addressed in the current study, existing research into the reasons for engaging in psychedelic experiences typically does not identify health behavior modification as a primary reason for use. Instead, common motives include physical/emotional healing and personal development, exploration of one’s consciousness and spirituality, and creativity enhancement^46–48^. The infrequent mentions surrounding health-related behaviors by participants (reported by practitioners), further suggest that health behaviors have not typically been a primary motivation for psychedelic use. However, since the early stages of clinical research, psychedelics have been explored as potential treatments for behavior-driven health conditions, such as alcohol use disorder^49^. Additionally, the potential of psychedelics to drive behavioral changes has been recognized as a key target for future research^50^ and also as a potential contributing factor for the success of psychedelic-assisted therapy^51^. Finally, it has been proposed that psychedelics hold transdiagnostic and prophylactic potential^52^. Although this framework has primarily been applied to the treatment of mental health conditions, its principles may also extend to the domain of health-related behaviors by opening a broader window for healthy change.

Guiding participants towards habits that support both preparation for, and integration of, psychedelic experiences are common practices among practitioners, which raises the question of whether health behavior-focused preparation and integration practices could enhance the potential for health behavior change following a psychedelic experience. Additionally, many of the changes in behaviors reported are part of the preparation and integration process facilitated by practitioners, and a carry-over effect from such practices in clients’ lives is possible. Incorporating reflection on health behaviors, and its inclusion in preparation and integration manuals, could enhance the beneficial effects of psychedelic experiences; however no studies have tested this directly. One partial exception is the qualitative study by Bathje and colleagues^46^ where participants indicated that previous engagement with the traditional “dieta” as a preparation practice facilitated the maintenance of a healthier diet after ayahuasca use. By explicitly including guidance on physical activity, meditation, mindfulness or other health-related behaviors in their protocols, practitioners could amplify the positive impact of these experiences on overall well-being and on behavioral and physical health.

When providing guidance to patients or clients, it is important to emphasize that healthy behavior changes may look different from person to person. For instance, in terms of diet and nutrition, a “healthier diet” may involve eating more or less of certain foods or nutrients, depending on individual needs. Similarly, for physical activity, some individuals might benefit from learning how to reduce it and rest effectively, which could have a positive impact on their overall fitness regimen in case they are overtraining. The findings of this study, with both increases and decreases in health behaviors reported, is aligned with this notion and reinforces the importance of tailoring guidance to each individual’s unique circumstances. As proposed by Neuhaus and Slavich^50^, psychedelic practitioners should incorporate the definition of behavioral goals in a personalized and individualized manner during the preparation phase.

For this study, we also intended to explore how psychedelic practitioners rated the importance of both well-established psychosocial mechanisms of behavior change (e.g., self-efficacy), some of which may also be considered as being particularly sensitive to psychedelic administration, such as psychological flexibility and the occurrence of psychological insights. Given both the limited number and variance in responses – where practitioners consistently rated almost all mechanisms as positively contributing to the association between psychedelic experiences and health behavior changes – it is challenging to expand on the relative role of these mechanisms based on the insights gained in the current study. It is possible that participants were not sufficiently knowledgeable about many or most of the concepts presented (despite the definition provided) to meaningfully opine on their relative role in behavior change. Future studies with more detailed or nuanced response options (e.g., broader scale ranges) or including qualitative methods, such as open-ended questions, semi-structured interviews, or thematic analysis, could provide a deeper exploration and help uncover practitioners’ perspective on different psychological mechanisms that may contribute to behavior change.

Although the present research cannot establish causality, our observations – both in terms of practitioner perceptions of others and report of their own behavior changes – support the hypothesis that psychedelics may facilitate shifts toward healthier living, even unintentionally, an assertion corroborated by different lines of evidence (reviewed before). Moreover, as suggested by the studies on ayahuasca users^23,24^ (vs. population data), psychedelic use may come to be seen as positive in promoting health and well-being and preventing disease throughout the lifespan^9,53^. However, it is important to note that some reports – both regarding clients/patients and practitioners’ own behaviors – indicated changes in a less healthy direction, highlighting the need to avoid assuming that psychedelic experiences are invariably health-promoting.

Given the nature of the present study, it is essential to account for the potential impact of self-selection bias. Practitioners who did not perceive behavior changes in their clients may have been less likely to participate. Relatedly, their own personal experiences with psychedelic substances and health behavior change could have influenced their perception regarding their client’s health behavior changes, potentially shaping the overall findings^54^.

Another limitation relates to the perception-based assessments of health behavior change. Given that changes in health behaviors were neither confirmed by the clients themselves, or their close family, nor supported by longitudinal data on clients’ actual health outcomes, we acknowledge the possibility of substantial mismatch between perceived and enacted behavior change. Self-reported perceptions from practitioners are subjective and could be influenced by their expectations regarding the effects of psychedelic experiences, their own personal experience with health behavior change associated with psychedelic use, or social norms (e.g., social desirability), which may not accurately reflect clients’ real-world behavior changes. In reporting both perceived changes in others and in their own behavior, practitioners with extensive personal and facilitation experience may be particularly well-positioned to provide informed perspectives. However, their extensive involvement – practitioners reported an average of 157 personal experiences – may also make them more susceptible to recall bias, which could influence and potentially skew their reports of behavior change. Additionally, although we were satisfied that 96 individuals chose to participate, they may not accurately represent the worldwide population of (likely) hundreds of formal and informal practitioners.

The shortage of demographic information is also a limitation. This could be attributed to the illegal status of psychedelics in many regions, which likely influenced participants’ reluctance to share personal information – even though anonymity was guaranteed in the study. It is plausible that many of the respondents were involved in underground psychedelic ceremonies or experiences, making them more cautious about revealing identifying details due to legal risks. Additionally, data on the setting and structure of each practitioners’ practice were not collected, limiting our ability to account for differences in practitioner-client interactions, retreat length, integration follow-up, or access to specific behaviors. These factors could influence both practitioners’ perceptions and clients’ actual behavior changes.

The present study contributes to our understanding of how psychedelic experiences may be related to health-related behavior changes. The findings suggest that, through the eyes of experienced practitioners, psychedelic experiences have an overall positive impact on health behaviors, such as physical activity, diet and nutrition, alcohol consumption, tobacco use and mindfulness practices, for some of those taking part in these sessions. Furthermore, practitioners report experiencing improvements in their own health-related behaviors, adopting lifestyle habits that undoubtedly contribute to overall health and well-being.

Given the initial phase of research addressing the association between psychedelic experiences and health behavior change, and the limitations inherent to this study, additional research is needed to clarify how, to what extent, and under which conditions psychedelic experiences may be used in the future as an effective tool, or as part of formal interventions, to facilitate positive behavior change.

## Method

### Design

This was a retrospective observational study, conducted through an anonymous online survey administered at a single time-point, with an average duration of 25 min. Participants were recruited through the online dissemination of the study webpage via various digital media, including social media platforms and psychedelic-related newsletters. Information regarding the study objectives, participation inclusion criteria, as well as anonymity and confidentiality were provided before participants were asked to give informed consent and proceed with the survey. Data was collected from May 2023 to May 2024.

Inclusion criteria required participants to have guided, supported or facilitated at least 30 psychedelic experiences (individual or group), and to have consumed a psychedelic substance at least 10 times. Participants also needed to be 18years old or older and have a sufficiently good understanding of the English language. The threshold for facilitation and personal use resulted from a discussion between the researchers on what might constitute “sufficient experience”, given that the study’s aim was to recruit experienced practitioners.

The study was approved by the Psychology Research Ethics Committee of Leiden University (reference number 4038) and was conducted in accordance with the Declaration of Helsinki^55^. No compensation or incentive offered to participants.

### Instrument

The questionnaire was specifically developed for this study by the research team and consisted of five sections: demographics and practitioners’ level/type of experience as a practitioner and user; changes in health-related behaviors perceived (by practitioners) in patients or clients; perceived frequency with which patients or clients mentioned these behavior changes; agreement with potential psychological mechanisms that helped explain the changes; and changes in practitioners own health-related behaviors in relation to (their own) past psychedelic experiences. The full survey is available in the Open Science Framework.

### Demographic information

The demographic information collected included age, sex, gender, country of origin, current country of residence, educational background, and employment status.

### Lifetime psychedelic use and lifetime experience as practitioner

Lifetime experience as a practitioner was assessed through the number of psychedelic sessions facilitated, the substances used during these sessions, and the primary psychedelic substance of choice.

Finally, participants reported the total number of psychedelic personal experiences they had within their lifetime, the substances they had used, and the primary substance used during these experiences (as a user).

### Frequency and direction of health behavior change in clients/patients

Participants were asked to report on changes that they perceived in their patients or clients in regards to the following health-related behaviors: physical activity, diet and nutrition, eating patterns/relation with food, alcohol consumption, tobacco use, caffeine consumption, cannabis use, other (non-psychedelic) drug use, psychiatric medication use (medically prescribed), non-prescribed psychiatric medication use, contemplative and/or mindfulness practices, time spent in nature, personally meaningful social activities, sleep, compliance with public health recommendations, screen use during leisure time, work-life balance, ice/cold bath or showers, and sauna use. Participants were also asked to indicate perceived changes in body weight. This set of behaviors encompasses those with well-established evidence of their impact on health and well-being (e.g., physical activity, tobacco use) as well as behaviors that could potentially influence health and well-being from the user’s perspective (e.g., ice/cold bath, sauna use). Including a broad range of behaviors is relevant in the early stages of research on the impact of psychedelic experiences as it allows for the exploration of emerging areas of interest in overall health and functioning.

Specifically, participants indicated the percentage of patients or clients for whom they perceived a change – either an increase or decrease – in the listed behaviors. For more complex health behaviors [note: by “complex health behaviors,” we refer to broader categories of behaviors that can be broken down into specific, observable actions. For example, physical activity includes specific behaviors such as walking, running, and dancing], when the overarching category was endorsed, specific subsets of behaviors were presented. For example, in the case of physical activity, after reporting the percentage of patients or clients for whom a change was perceived, participants were presented with a list of specific physical activities (e.g., hiking, running). For each specific physical activity, participants indicated the direction of the change (increased or decreased) perceived. This procedure offers a more detailed evaluation of changes in the following (complex) health-related behaviors: physical activity, diet and nutrition, eating patterns/relation with food, contemplative and/or mindfulness practices, time spent in nature, personally meaningful social activities and work-life balance.

### Mentions of behavior changes by patients or clients before, during and after sessions

Participants were asked to report how frequently health-related behavior changes were mentioned by their patients or clients, for each of the behaviors presented, during the three phases of the psychedelic experience: preparation, the psychedelic experience itself and integration. Response options on a 4-point scale ranged from rarely (0–25% of the time) to very often (76–100% of the time).

### Psychological mechanisms for health behavior change

Based in scientifically established psychological mechanism underlying behavior change (Carey et al., 2019; Hagger et al., 2020) and informed by the current literature on the potential mechanisms related to psychedelic effects (Kočárová et al., 2021; van Elk & Yaden, 2022), the psychosocial variables presented were: self-efficacy, autonomy, competence, relatedness, open-heartedness, health as an identity, health as an inspiration, psychological flexibility, integrated emotion regulation, mystical experience, awe, quieted ego/ego dissolution, self-compassion, bodily awareness, mortality acceptance, preparation, integration, self-criticism, values and committed action, subjective/psychological well-being, coherence/self-concordance, intrinsic-extrinsic values, perceived good-life coherence, sense of purpose in life, connection to nature, *communitas*, emotional breakthrough, psychological insight, and intuitive knowledge. For each psychological mechanism, participants indicated their level of agreement regarding the strength of the association between the psychological mechanism and health behavior change, using a five-point scale from strongly disagree to strongly agree. An attention check item was included to account for inadvertent or careless responses, and each mechanism was accompanied by a brief definition, intended to reduce literacy bias and support consistent interpretation across participants (see Supplementary File_3).

### Practitioners’ own health behavior change

For each behavior, practitioners first indicated whether a change was experienced and then specified the direction of that change. The option “No, and I did not engage with this behavior before my psychedelic experience(s)” was available for the following health-related behaviors: alcohol consumption, tobacco use, caffeine consumption, cannabis use, other drug use, psychiatric medication, non-prescribed psychiatric medication, contemplative and/or mindfulness practices, ice/cold bath or shower and sweat lodge/sauna use. Secondly, if participants reported a change in a complex behavior, they were presented with a list of subsets of specific behaviors and asked to indicate the direction of change for the specific behaviors where a change was experienced.

### Data analysis

Descriptive analyses were conducted to summarize practitioners’ perceptions of health behavior changes in their clients following psychedelic experiences. Frequency distributions and percentages were calculated for categorical variables, such as the proportion of practitioners reporting increases, decreases, or no change in each behavior. Means and standard deviations were computed for continuous variables, including the percentage of clients (out of the total each practitioner had worked with) for whom a given change was perceived. No inferential statistical tests were performed, as the study aimed to provide an overview of practitioners’ perceptions rather than test specific hypotheses. All analyses were conducted using the IBM SPSS statistical software version 29.0 (https://www.ibm.com/products/spss-statistics).

## Supplementary Information

Below is the link to the electronic supplementary material.

Supplementary Material 1

## Data Availability

The data that support the findings of this study are available from the corresponding author (PJT), upon reasonable request. The study was registered in the Open Science Framework.

## References

[1] Hacker, K. The burden of chronic disease. *Mayo Clin. Proceedings: Innovations Qual. Outcomes*. **8** (1), 112–119 (2024). 10.1016/j.mayocpiqo.2023.08.005 PMC10830426 38304166

[2] *World Health Organization* (2024). https://www.who.int/news-room/fact-sheets/detail/noncommunicable-diseases

[3] *World Health Organization* (2025). https://www.who.int/news-room/fact-sheets/detail/obesity-and-overweight

[4] *World Health Organization* (2025). https://www.who.int/news-room/fact-sheets/detail/tobacco

[5] *World Health Organization* (2024). https://www.who.int/news-room/fact-sheets/detail/physical-activity

[6] Michie, S., van Stralen, M. M. & West, R. The behaviour change wheel: A new method for characterising and designing behaviour change interventions. *Implement. Sci.* **6** (42), 12–19 (2011). 21513547 10.1186/1748-5908-6-42 PMC3096582

[7] Michie, S., Atkins, L. & West, R. *The Behavior Change Wheel: A Guide To Designing Interventions* (Silverback Publishing, 2014).

[8] Hagger, M. S., Cameron, L. D., Hamilton, K., Hankonen, N. & Lintunen, T. The science of behavior change: the road ahead in The Handbook of Behavior Change (eds Hagger, M. S., Cameron, L. D., Hamilton, K., Hankonen, N. & Lintunen, T.) 677–699 (Cambridge University Press, (2020).

[9] Teixeira, P. J. et al. Psychedelics and health behaviour change. *J. Psychopharmacl*. **36** (1), 12–19 (2022). 10.1177/02698811211008554 PMC8801670 34053342

[10] Bogenschutz, M. P. et al. Percentage of heavy drinking days following Psilocybin-Assisted psychotherapy vs placebo in the treatment of adult patients with alcohol use disorder. *JAMA Psychiatry. ***79**(10), 953-962 (2022). 36001306 10.1001/jamapsychiatry.2022.2096 PMC9403854

[11] Berlowitz, I., Walt, H., Ghasarian, C., Mendive, F. & Martin-Soelch, C. Short-Term treatment effects of a substance use disorder therapy involving traditional Amazonian medicine. *J. Psychoact. Drugs*. **51**, 323–334 (2019). 10.1080/02791072.2019.1607956 31043116

[12] Garcia-Romeu, A. et al. Cessation and reduction in alcohol consumption and misuse after psychedelic use. *J. Psychopharmacol.* **33**, 1088–1101 (2019). 31084460 10.1177/0269881119845793

[13] Perkins, D. et al. Associations between Ayahuasca consumption in naturalistic settings and current alcohol and drug use: results of a large international cross-sectional survey. *Drug Alcohol Rev.* **41**, 265–274 (2022). 34308566 10.1111/dar.13348

[14] Thomas, G., Lucas, P., Rielle Capler, N., Tupper, K. W. & Martin, G. Ayahuasca-Assisted therapy for addiction: results from a preliminary observational study in Canada. *Curr. Drug Abuse Rev.* **6** (1), 30–42 (2013). 23627784 10.2174/15733998113099990003

[15] Johnson, M. W., Garcia-Romeu, A., Cosimano, M. P. & Griffiths, R. R. Pilot study of the 5-HT2AR agonist psilocybin in the treatment of tobacco addiction. *J. Psychopharmacol.* **28**, 983–992 (2014). 25213996 10.1177/0269881114548296 PMC4286320

[16] Daldegan-Bueno, D., Maia, L. O., Massarentti, C. M. & Tófoli, L. F. Ayahuasca and tobacco smoking cessation: results from an online survey in Brazil. *Psychopharmacology* **239**, 1767–1782 (2022). 35179623 10.1007/s00213-022-06063-2

[17] Johnson, M. W., Garcia-Romeu, A. & Griffiths, R. R. Long-term follow-up of psilocybin-facilitated smoking cessation. *Am. J. Drug Alcohol Abuse*. **43**, 55–60 (2017). 27441452 10.3109/00952990.2016.1170135 PMC5641975

[18] Johnson, M. W., Garcia-Romeu, A., Johnson, P. S. & Griffiths, R. R. An online survey of tobacco smoking cessation associated with naturalistic psychedelic use. *J. Psychopharmacol.* **31**, 841–850 (2017). 28095732 10.1177/0269881116684335 PMC6753943

[19] Jones, G., Lipson, J. & Nock, M. K. Associations between classic psychedelics and nicotine dependence in a nationally representative sample. *Sci Rep* **12**, (2022). 10.1038/s41598-022-14809-3 PMC9216303 35732796

[20] Simonsson, O., Osika, W., Carhart-Harris, R. & Hendricks, P. S. Associations between lifetime classic psychedelic use and cardiometabolic diseases. *Sci Rep* **11**, (2021). 10.1038/s41598-021-93787-4 PMC8277805 34257396

[21] Simonsson, O., Sexton, J. D. & Hendricks, P. S. Associations between lifetime classic psychedelic use and markers of physical health. *J. Psychopharmacol.* **35**, 447–452 (2021). 33719688 10.1177/0269881121996863 PMC8056715

[22] Simonsson, O., Hendricks, P. S., Chambers, R., Osika, W. & Goldberg, S. B. Classic psychedelics, health behavior, and physical health. *Ther Adv. Psychopharmacol* **12**, (2022). 10.1177/20451253221135363 PMC9716448 36465958

[23] Kohek, M. et al. Ayahuasca and public health II: health status in a large sample of Ayahuasca-Ceremony participants in the Netherlands. *J. Psychoact. Drugs*. **55** (3), 1–12 (2022). 10.1080/02791072.2022.2077155 35635152

[24] Ona, G. et al. Ayahuasca and public health: health Status, psychosocial Well-Being, Lifestyle, and coping strategies in a large sample of ritual Ayahuasca users. *J. Psychoact. Drugs*. **51**, 135–145 (2019). 10.1080/02791072.2019.1567961 30732540

[25] Nichols, D. E. & Psychedelics *Pharmacol. Rev.* **68**(2), 264–355 (2016). 26841800 10.1124/pr.115.011478 PMC4813425

[26] *Booking.com* (2023). https://www.booking.com/c/trends/travelpredictions2024.html?msockid=21ab7b2ced05617518ce6e5cec6a60ba

[27] *Retreat Guru* https://retreat.guru/.

[28] Hillier, D. Hedonism, healing and credible science – where next for drug tourism? (2024). https://roadbook.com/opinion/drug-tourism-retreats-health-hedonism/

[29] Winstock, A. R. et al. Global Drug Survey (GDS) 2020: Psychedelics key findings report (2020). https://www.globaldrugsurvey.com/gds-2020-psychedelics-report/

[30] Barnett, B. S., Siu, W. O. & Pope, H. G. A survey of American psychiatrists’ attitudes toward classic hallucinogens. *J. Nerv. Mental Disease*. **206**, 476–480 (2018). 10.1097/NMD.0000000000000828 29781894

[31] Davis, A. K., Agin-Liebes, G., España, M., Pilecki, B. & Luoma, J. Attitudes and beliefs about the therapeutic use of psychedelic drugs among psychologists in the united States. *J. Psychoact. Drugs*. **54** (4), 309–318 (2021). 10.1080/02791072.2021.1971343 34468293

[32] Downey, A. E., Boyd, M., Chaphekar, A. V., Woolley, J. & Raymond-Flesch, M. But the reality is it’s happening: A qualitative study of eating disorder providers about psilocybin-assisted psychotherapy. *Int. J. Eat. Disord.* **56** (11), 2142–2148 (2023). 37551650 10.1002/eat.24041 PMC12965192

[33] Kraiem, E., Diener, M., Guss, J., Mavrides, L. & Saban, S. Psychoanalyst attitudes towards psychedelic-assisted therapy. *Drugs: Educ. Prev. Policy.* **32**(5), 449-460 (2024).

[34] Meir, P., Taylor, L., Soares, J. C. & Meyer, T. D. Psychotherapists’ openness to engage their patients in Psilocybin-Assisted therapy for mental health treatment. *J. Affect. Disord*. **323**, 748–754 (2023). 36535547 10.1016/j.jad.2022.12.050

[35] Sholevar, R., Peteet, J., Sanders, J. & Beaussant, Y. Disruption as an opportunity or threat: A qualitative analysis of factors influencing the attitudes of experts in serious illness care toward psychedelic-assisted therapies. *Palliat. Support Care*. **21** (6), 967–972 (2023). 37818641 10.1017/S1478951523001475

[36] Wells, A., Fernandes, M. & Reynolds, L. Perceptions and attitudes towards psychedelic-assisted psychotherapy among health professionals, patients, and the public: A systematic review. *J. Psychedelic Stud.* **8**, 43–62 (2024).

[37] Callon, C., Williams, M. & Lafrance, A. Meeting the medicine halfway: Ayahuasca ceremony leaders’ perspectives on Preparation and integration practices for participants. *J Humanist Psychol. ***0**(0), 1–27 (2021).

[38] Earleywine, M., Low, F., Lau, C. & De Leo, J. Integration in Psychedelic-Assisted treatments: recurring themes in current providers’ Definitions, Challenges, and concerns. *J Humanist Psychol. ***0**(0), 1–18 (2022).

[39] Williams, M., Kingston Miller, A., Loizaga-Velder, A., Files, N. & Lafrance, A. Getting to the root: Ayahuasca ceremony leaders’ perspectives on eating disorders. *J. Psychoact. Drugs*. **55** (4), 425–433 (2023). 10.1080/02791072.2022.2113484 36171638

[40] Glynos, N. G. et al. Going underground: Demographics, Services, and best practices endorsed by practitioners providing support for naturalistic psychedelic use. *J Psychoact. Drugs*, 1–11 (2024). 10.1080/02791072.2024.2405685 PMC11919790 39297183

[41] Hughes, S. et al. Psilocybin mushroom stewardship: A qualitative inquiry into practices and priorities of underground psilocybin mushroom practitioners. *J Psychedelic Stud.*** 9**(3), 221-233 (2025).

[42] Watts, R., Day, C., Krzanowski, J., Nutt, D. & Carhart-Harris, R. Patients’ accounts of increased connectedness and acceptance after psilocybin for Treatment-Resistant depression. *J. Humanist Psychol.* **57**, 520–564 (2017).

[43] Agin-Liebes, G. I. et al. Long-term follow-up of psilocybin-assisted psychotherapy for psychiatric and existential distress in patients with life-threatening cancer. *J. Psychopharmacol.* **34**, 155–166 (2020). 31916890 10.1177/0269881119897615

[44] Garcia-Romeu, A. et al. Persisting reductions in Cannabis, Opioid, and stimulant misuse after naturalistic psychedelic use: an online survey. *Front Psychiatry.* **10**, (2020). 10.3389/fpsyt.2019.00955 PMC6987443 32038317

[45] Raison, C. L., Jain, R., Penn, A. D., Cole, S. P. & Jain, S. Effects of naturalistic psychedelic use on Depression, Anxiety, and Well-Being: associations with patterns of use, reported Harms, and transformative mental States. *Front Psychiatry* **13**, (2022). 10.3389/fpsyt.2022.831092 PMC8965278 35370864

[46] Bathje, G. J., Fenton, J., Pillersdorf, D. & Hill, L. C. A qualitative study of intention and impact of Ayahuasca use by Westerners. *J. Humanist Psychol.* **64**, 653–691 (2024).

[47] Pestana, J., Beccaria, F. & Petrilli, E. Psychedelic substance use in the Reddit psychonaut community. A qualitative study on motives and modalities. *Drugs Alcohol Today*. **21**, 112–123 (2020).

[48] Roberts, C. A., Osborne-Miller, I., Cole, J., Gage, S. H. & Christiansen, P. Perceived harm, motivations for use and subjective experiences of recreational psychedelic ‘magic’ mushroom use. *J. Psychopharmacol.* **34**, 999–1007 (2020). 32674668 10.1177/0269881120936508

[49] Krebs, T. S., Johansen, P. & Ør Lysergic acid diethylamide (LSD) for alcoholism: Meta-analysis of randomized controlled trials. *J. Psychopharmacol.* **26** (7), 994–1002 (2012). 22406913 10.1177/0269881112439253

[50] Neuhaus, E. C. & Slavich, G. M. Behavioral psychedelics: integrating Mind and behavior to improve health and resilience. *Front Psychiatry* **13**, (2022). 10.3389/fpsyt.2022.821208 PMC8964031 35360129

[51] van Elk, M. & Yaden, D. B. Pharmacological, neural, and psychological mechanisms underlying psychedelics: A critical review. *Neuroscience Biobehavioral Reviews* **140**, (2022). 10.1016/j.neubiorev.2022.104793 35878791

[52] Kočárová, R., Horáček, J. & Carhart-Harris, R. Does psychedelic therapy have a transdiagnostic action and prophylactic potential? *Frontiers Psychiatry* **12** (2021). 10.3389/fpsyt.2021.661233 PMC8327748 34349678

[53] Kuiper, H. et al. Psychedelic public health: state of the field and implications for equity. *Soc Sci. Med* **357**, (2024). 10.1016/j.socscimed.2024.117134 39173415

[54] van Elk, M. & Fried, E. I. History repeating: guidelines to address common problems in psychedelic science. *Ther Adv. Psychopharmacol.* **13**, 1-20 (2023). 10.1177/20451253231198466 PMC10521293 37766730

[55] World Medical Association. World medical association declaration of helsinki: ethical principles for medical research involving human subjects. *JAMA* **310**, 2191–2194 (2013). 24141714 10.1001/jama.2013.281053

