# Correction: Exploring practitioners’ perceptions of health behavior changes associated with psychedelic experiences

**DOI:** 10.1038/s41598-025-33296-w

**Published:** 2025-12-29

**Authors:** Laura C. Carvalho, Jorge Encantado, Michiel van Elk, Arlen C. Moller, Talea Cornelius, Christopher Timmermann, Diogo Veiga, Pedro J. Teixeira

**Affiliations:** 1 Centro Interdisciplinar para o Estudo da Performance Humana (CIPER), Faculty of Human Kinetics, University of Lisbon, Lisbon, Portugal; 2 Cognitive Psychology Unit, Leiden University, Leiden, Netherlands; 3 Department of Psychology, Illinois Institute of Technology, Chicago, USA; 4 Center for Behavioral Cardiovascular Health, Columbia University Irving Medical Center, New York, USA; 5 Department of Experimental Psychology, UCL, London, UK; 6 DMT Research Group, Department of Brain Sciences, Imperial College London, London, UK

Correction to: *Scientific Reports* 10.1038/s41598-025-25818-3, published online 25 November 2025

The original version of this Article contained errors. Figure 1 displayed an incomplete version, where the behaviors ‘Diet and nutrition’, ‘Alcohol consumption’, ‘Social activities’, ‘Time spent in nature’, ‘Cannabis use’, ‘Caffeine consumption’, ‘Work-life balance’, ‘Screen use’, ‘Public health’, and ‘Non-prescribed psychiatric medication’, and their respective scores were omitted. The original Figure 1 and accompanying legend appear below.

**Fig. 1:**
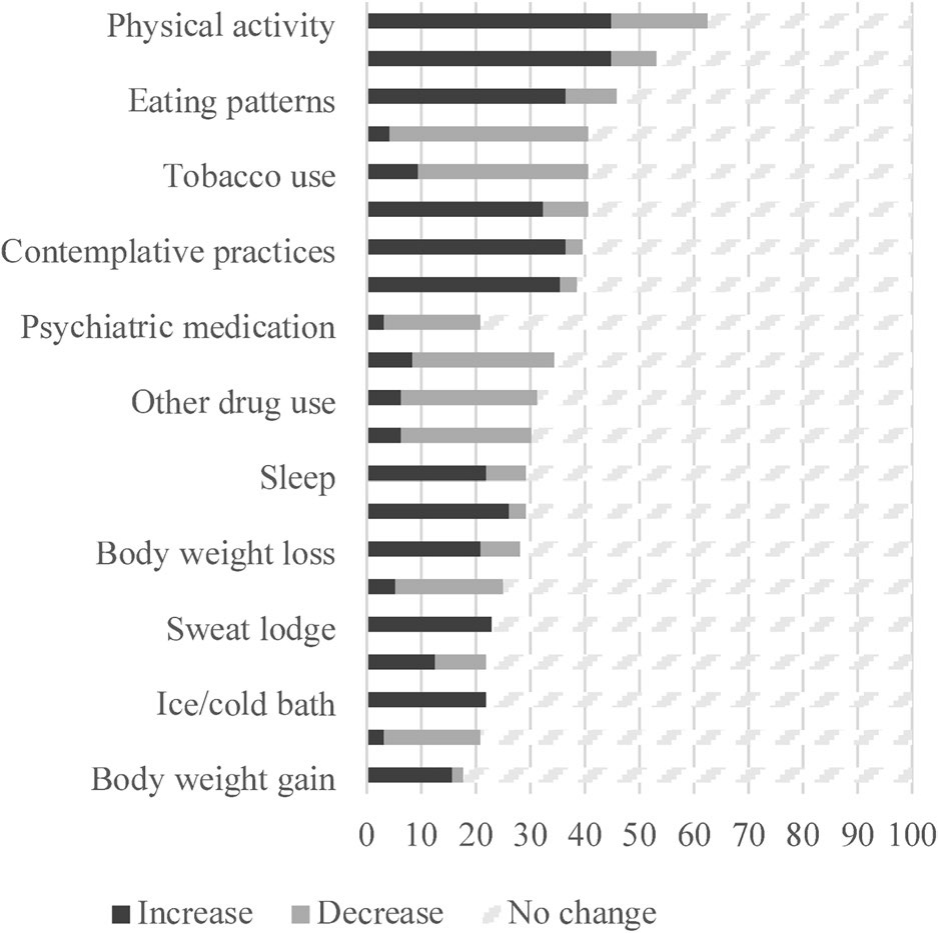
Percentage of practitioners reporting changes in clients’ or patients’ health-related behaviors (*n* = 96).

In addition, Figure 2 displayed an incomplete version, where the psychological mechanisms ‘Integration’, ‘Preparation’, ‘Psychological flexibility’, ‘Integrated emotional regulation’, ‘Open-heartedness’, ‘Emotional breakthrough’, ‘Psychological insight’, ‘Awe’, ‘Autonomy’, ‘Intuitive knowledge’, ‘Intrinsic-extrinsic values’, ‘Subjective/psychological well-being’, ‘Competence’ and ‘Health as an identity’, and their respective scores were omitted. The original Figure 2 and accompanying legend appear below.

**Fig. 2:**
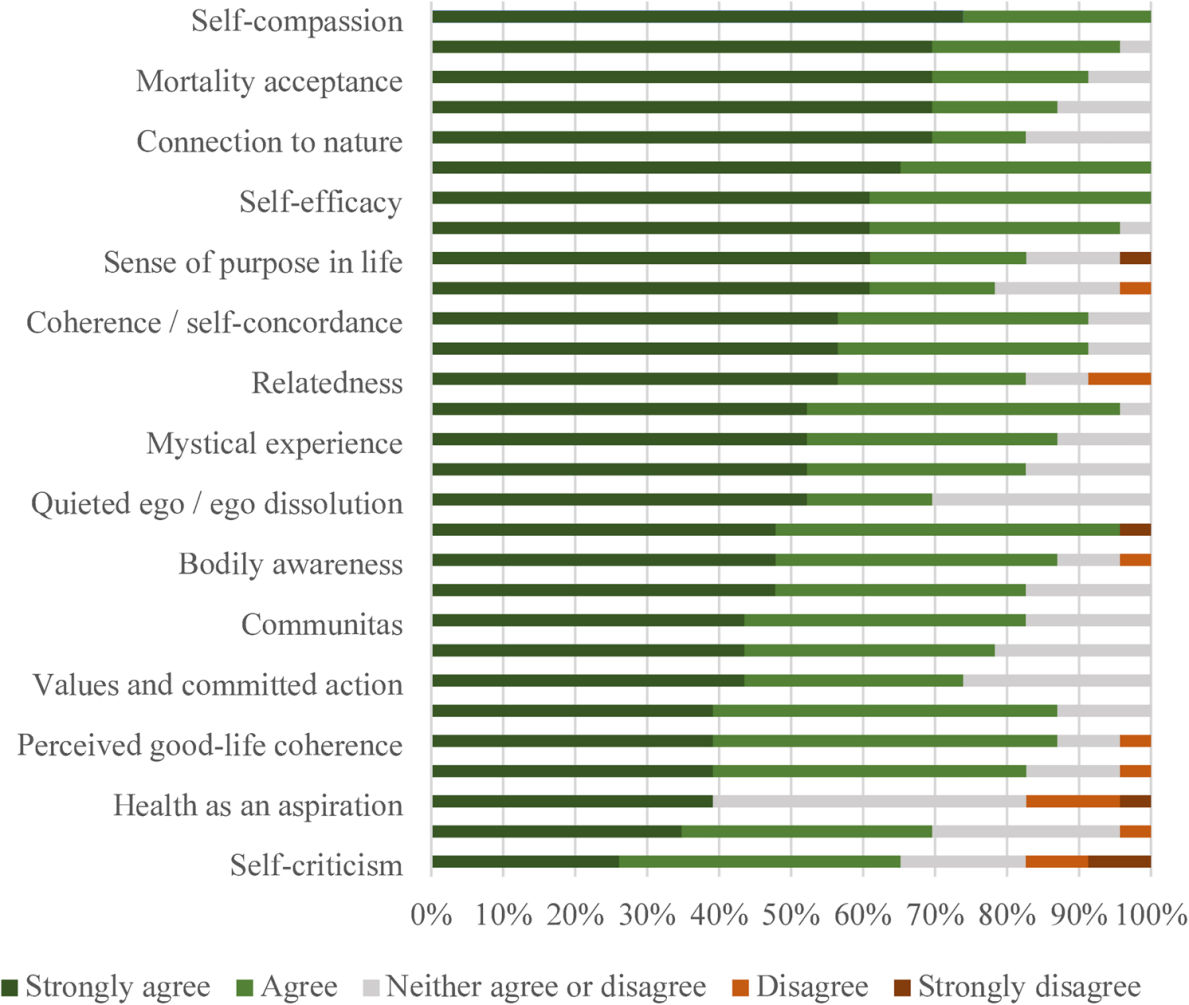
Association between psychological mechanisms and health behavior change (*n* = 23).

The original Article has been corrected.

