# Supplementary material for: Exploring practitioners’ perceptions of health behavior changes associated with psychedelic experiences
Source: Sci Rep. 2025 Nov 25;15:41812. doi: 10.1038/s41598-025-25818-3 (PMC12647726; doi:10.1038/s41598-025-25818-3)
Supplement: Supplementary file 1 — Supplementary Material 1 [file 41598_2025_25818_MOESM1_ESM.docx]

**Supplementary File 1**

***Perceived changes in clients’ or patients’ main health-related behaviors (reported by practitioners)***

| ***Health Behaviors*** |  | **N** | **%** | **Mean % (SD) of clients** |
| --- | --- | --- | --- | --- |
| Physical activity (n = 96) | Change | 60 | 62.5% |  |
|  | *Increased* | 43 | 44.8% | 46.7% (25.1) |
|  | *Decreased* | 17 | 17.7% | 24% (27.8) |
|  | No change | 36 | 37.5% |  |
| Diet and nutrition (n = 96) | Change | 51 | 53.1% |  |
|  | *Healthier* | 43 | 44.8% | 56% (23.5) |
|  | *Less healthy* | 8 | 8.3% | 25.1% (28.7) |
|  | No change | 45 | 46.9% |  |
| Eating patterns (n = 96) | Change | 44 | 45.8% |  |
|  | *Healthier* | 35 | 36.5% | 56.9% (26.4) |
|  | *Less healthy* | 9 | 9.4% | 22.3% (29.1) |
|  | No change | 52 | 54.2% |  |
| Body weight loss (n = 96) | Change | 27 | 28.1% |  |
|  | *Healthy* | 20 | 20.8% | 40.9% (28.4) |
|  | *Unhealthy* | 7 | 7.3% | 18.4% (22.4) |
|  | No change | 69 | 71.9% |  |
| Body weight gain (n = 96) | Change | 17 | 17.7% |  |
|  | *Healthy* | 15 | 15.6% | 40.2% (28.8) |
|  | *Unhealthy* | 2 | 2.1% | 39.5% (51.6) |
|  | No change | 79 | 82.3% |  |
| Alcohol consumption (n = 96) | Change | 39 | 40.6% |  |
|  | *Increased* | 4 | 4.2% | 42.3% (42.7) |
|  | *Decreased* | 35 | 36.5% | 53.1% (26.2) |
|  | No change | 57 | 59.4% |  |
| Tobacco use (n = 96) | Change | 39 | 40.6% |  |
|  | *Increased* | 9 | 9.4% | 23.2% (23.8) |
|  | *Decreased* | 30 | 31.3% | 47.2% (29.6) |
|  | No change | 57 | 59.4% |  |
| Caffeine consumption (n = 96) | Change | 29 | 30.2% |  |
|  | *Increased* | 6 | 6.3% | 38.5% (35) |
|  | *Decreased* | 23 | 24% | 44.3% (23.2) |
|  | No change | 67 | 69.8% |  |
| Cannabis use (n = 96) | Change | 33 | 34.4% |  |
|  | *Increased* | 8 | 8.3% | 35.4% (21.1) |
|  | *Decreased* | 25 | 26% | 33% (21.4) |
|  | No change | 63 | 65.6% |  |
| Other drug use (n = 96) | Change | 30 | 31.3% |  |
|  | *Increased* | 6 | 6.3% | 17% (14.8) |
|  | *Decreased* | 24 | 25% | 60.8% (27.7) |
|  | No change | 66 | 68.8% |  |
| Psychiatric med. use (n = 96) | Change | 34 | 35.4% |  |
|  | *Increased* | 7 | 7.3% | 12.4% (8.9) |
|  | *Decreased* | 27 | 28.1% | 53.4% (31.8) |
|  | No change | 62 | 64.6% |  |
| Non-prescribed med use (n = 96) | Change | 20 | 20.8% |  |
|  | *Increased* | 3 | 3.1% | 33.7% (36.1) |
|  | *Decreased* | 17 | 17.7% | 44.5% (34.3) |
|  | No change | 76 | 79.2% |  |
| Contemplative practices (n = 96) | Change | 38 | 39.6% |  |
|  | *Increased* | 35 | 36.5% | 61.4% (24.2) |
|  | *Decreased* | 3 | 3.1% | 6% (1.0) |
|  | No change | 58 | 60.4% |  |
| Time spent in nature (n = 96) | Change | 37 | 38.5% |  |
|  | *Increased* | 34 | 35.4% | 60.1% (27.6) |
|  | *Decreased* | 3 | 3.1% | 17.7% (14.5) |
|  | No change | 59 | 61.5% |  |
| Personally meaningful social activities (n = 96) | Change | 39 | 40.6% |  |
|  | *Increased* | 31 | 32.3% | 55.2% (25.6) |
|  | *Decreased* | 8 | 8.3% | 26.6% (12.3) |
|  | No change | 57 | 59.4% |  |
| Sleep quality (n = 96) | Change | 28 | 29.2% |  |
|  | *Increased* | 21 | 21.9% | 50.3% (28.0) |
|  | *Decreased* | 7 | 7.3% | 30.6% (16) |
|  | No change | 68 | 70.8% |  |
| Compliance public health recommendations (n = 96) | Change | 21 | 21.9% |  |
|  | *Increased* | 12 | 12.5% | 35.1% (24.2) |
|  | *Decreased* | 9 | 9.4% | 36.4% (20.9) |
|  | No change | 75 | 78.1% |  |
| Screen use (n = 96) | Change | 24 | 25% |  |
|  | *Increased* | 5 | 5.2% | 21.8% (23.8) |
|  | *Decreased* | 19 | 19.8% | 47.2% (25.4) |
|  | No change | 72 | 75% |  |
| Work-life balance (n = 96) | Change | 28 | 29.2% |  |
|  | *Increased* | 25 | 26% | 43.7% (26.1) |
|  | *Decreased* | 3 | 3.1% | 29.3% (24.0) |
|  | No change | 68 | 70.8% |  |
| Ice/cold bath or shower (n = 96) | Change | 21 | 21.9% |  |
|  | *Increased* | 21 | 21.9% | 32.7% (27.1) |
|  | *Decreased* | 0 | 0% | - |
|  | No change | 75 | 78.1% |  |
| Sweat lodge/sauna use (n = 96) | Change | 22 | 22.9% |  |
|  | *Increased* | 22 | 22.9% | 40% (29.7) |
|  | *Decreased* | 0 | 0% | - |
|  | No change | 74 | 77.1% |  |

***Perceived changes in clients’ or patients’ specific health-related behaviors (reported by practitioners)***

| ***Health Behaviors*** | | **Total change** | **Increased** | **Decreased** |
| --- | --- | --- | --- | --- |
|  |  | **%** | **%** | **%** |
| *Physical activity (n = 60)* | Yoga (n = 34) | 56.7% | 55% | 1.04% |
|  | Dancing (n = 31) | 51.7% | 51.7% | 0% |
|  | Walking (n = 31) | 51.7% | 50% | 1.67% |
|  | Hiking (n = 26) | 43.3% | 41.7% | 1.67% |
|  | Running (n = 22) | 36.7% | 33.3% | 3.33% |
|  | Swimming (n = 19) | 31.7% | 26.7% | 5% |
|  | Cycling (n = 18) | 30% | 28.3% | 1.67% |
|  | Physical exercise (n = 18) | 30% | 28.3% | 1.67% |
|  | Martial arts (n = 16) | 26.7% | 23.3% | 3.33% |
|  | Pilates (n = 14) | 23.3% | 23.3% | 0% |
|  | Team sports (n = 9) | 15% | 13.3% | 1.67% |
| *Diet and nutrition (n = 51)* | Vegetables and fruits (n = 39) | 76.5% | 76.5% | 0% |
|  | Consumption of sugar-based foods and drinks (n = 35) | 68.6% | 0% | 68.6% |
|  | Consumption of vegan/vegetarian meals (n = 35) | 68.6% | 64.7% | 3.92% |
|  | Consumption of processed foods (n = 31) | 60.8% | 0% | 60.8% |
|  | Consumption of meat (n = 31) | 60.8% | 5.88% | 54.9% |
|  | Consumption of nuts and whole grains (n = 26) | 51% | 51% | 0% |
|  | Consumption of legumes (n = 23) | 45.1% | 43.1% | 1.96% |
| *Eating patterns (n =44)* | Eating according to one’s body needs (n = 29) | 65.9% | 65.9% | 0% |
|  | Slow, mindful eating (n = 26) | 59.1% | 59.1% | 0% |
|  | Binges and cravings (n = 25) | 56.8% | 4.55% | 52.3% |
|  | Flexible eating (n = 24) | 54.5% | 50% | 4.55% |
|  | Enjoyment of a broader range of foods without guilt (n = 22) | 50% | 47.7% | 2.27% |
|  | Purgative behaviors (n = 20) | 45.5% | 11.4% | 34.1% |
|  | Ability to balance calories in vs calories out (n = 16) | 36.4% | 36.4% | 0% |
| Personally meaningful social activities *(n = 39)* | Social gatherings (n = 27) | 69.2% | 69.2% | 0% |
|  | Community engagement (n = 26) | 66.7% | 64.1% | 2.56% |
|  | Joining a group (n = 24) | 61.5% | 59% | 2.56% |
|  | Religious services (n = 17) | 43.6% | 28.2% | 15.4% |
|  | Volunteering (n = 15) | 38.5% | 33.3% | 5.13% |
|  | Political participation (n = 14) | 35.9% | 23.1% | 12.8% |
| *Contemplative practices (n = 38)* | Meditation (n = 35) | 92.1% | 92.1% | 0% |
|  | Mindfulness (n = 33) | 86.8% | 86.8% |  |
|  | Spiritual practices (n = 31) | 81.6% | 81.6% |  |
|  | Stress reduction practices (n = 27) | 71.1% | 68.4% | 2.63% |
|  | Prayer (n = 26) | 68.4% | 63.2% | 5.26% |
| *Time spent in nature (n = 37)* | Of “high quality” time spent in nature (n = 29) | 78.4% | 78.4% | 0% |
|  | In surrounding greenspace (n = 29) | 78.4% | 78.4% | 0% |
|  | In urban greenspace (n = 25) | 67.6% | 67.6% | 0% |
|  | In surrounding bluespace ( 24) | 64.9% | 64.9% | 0% |
|  | In urban bluespace (n = 24) | 64.9% | 64.9% | 0% |
|  | Gardening (n = 19) | 51.4% | 51.4% | 0% |
| *Work-life balance (n = 28)* | Reducing time spent working (n = 23) | 82.1% | 78.6% | 3.57% |
|  | Not working during leisure hours (n = 22) | 78.6% | 78.6% | 0% |
|  | Scheduling limits (n = 22) | 78.6% | 75% | 3.57% |

**Supplementary File 2**

***Main health-related behavior changes reported by practitioners (from their own experiences)***

| ***Health Behaviors*** |  | **N** | **%** |
| --- | --- | --- | --- |
| Contemplative practices (n = 30) | Change | 27 | 93.1% |
|  | No change | 2 | 6.9% |
| I did not engage in this behavior before | | 1 | 3.3% |
| Time spent in nature (n = 30) | Change | 26 | 86.7% |
|  | No change | 4 | 13.3% |
| Eating patterns (n = 30) | Change | 25 | 83.3% |
|  | No change | 5 | 16.7% |
| Personally meaningful social activities (n = 29) | Change | 24 | 82.8% |
|  | No change | 5 | 17.2% |
| Diet and nutrition (n = 32) | Change | 26 | 81.3% |
|  | No change | 6 | 18.8% |
| Alcohol consumption (n = 30) | Change | 15 | 78.9% |
|  | *Increased* | 0 | 0% |
|  | *Decreased* | 15 | 78.9% |
|  | No change | 4 | 21.1% |
| I did not engage in this behavior before | | 11 | 36.7% |
| Tobacco use (n = 25) | Change | 13 | 76.5% |
|  | *Increased* | 5 | 29.4% |
|  | *Decreased* | 8 | 47.1% |
|  | No change | 4 | 23.5% |
| I did not engage in this behavior before | | 8 | 26.7% |
| Physical activity (n = 32) | Change | 24 | 75% |
|  | No change | 8 | 25% |
| Work-life balance (n = 29) | Change | 19 | 73.1% |
|  | No change | 7 | 26.9% |
| Does not apply to my professional situation | | 3 | 10.3% |
| Cannabis use (n = 30) | Change | 12 | 63.2% |
|  | *Increased* | 3 | 15.8% |
|  | *Decreased* | 9 | 47.4% |
|  | No change | 7 | 36.8% |
| I did not engage in this behavior before | | 11 | 36.7% |
| Ice/cold bath or shower (n = 29) | Change | 12 | 60% |
|  | *Increased* | 12 | 60% |
|  | *Decreased* | 0 | 0% |
|  | No change | 8 | 40% |
| I did not engage in this behavior before | | 9 | 31% |
| Psychiatric medication use (n = 30) | Change | 4 | 57.1% |
|  | *Increased* | 0 | 0% |
|  | *Decreased* | 4 | 57.1% |
|  | No change | 3 | 42.9% |
| I did not engage in this behavior before | | 23 | 76.7% |
| Sweat lodge/sauna use (n = 29) | Change | 13 | 50% |
|  | *Increased* | 13 | 50% |
|  | *Decreased* | 0 | 0% |
|  | No change | 13 | 50% |
| I did not engage in this behavior before | | 3 | 10.3% |
| Screen use (n = 29) | Change | 14 | 48.3% |
|  | *Increased* | 0 | 0% |
|  | *Decreased* | 14 | 100% |
|  | No change | 15 | 51.7% |
| Sleep quality (n = 29) | Change | 12 | 41.4% |
|  | *Increased quality* | 10 | 83.3% |
|  | *Decreased quality* | 2 | 16.7% |
|  | No change | 17 | 58.6% |
| Other drug use (n = 30) | Change | 4 | 40% |
|  | *Increased* | 1 | 10% |
|  | *Decreased* | 3 | 30% |
|  | No change | 6 | 60% |
| I did not engage in this behavior before | | 20 | 66.7% |
| Compliance with public health recommendations (n = 29) | Change | 11 | 37.9% |
|  | *Increased* | 9 | 81.8% |
|  | *Decreased* | 2 | 18.2% |
|  | No change | 18 | 62.1% |
| Caffeine consumption (n = 30) | Change | 8 | 36.4% |
|  | *Increased* | 1 | 4.5% |
|  | *Decreased* | 7 | 31.8% |
|  | No change | 14 | 63.6% |
| I did not engage in this behavior before | | 8 | 26.7% |
| Non-prescribed medication use (n = 30) | Change | 2 | 28.6% |
|  | *Increased* | 0 | 0% |
|  | *Decreased* | 2 | 28.6% |
|  | No change | 5 | 71.4% |
| I did not engage in this behavior before | | 23 | 76.7% |
| Body weight (n = 30) | Change | 8 | 26.7% |
|  | No change | 22 | 73.3% |

***Specific health-related behavior changes reported by practitioners (from their own experiences)***

| ***Health behaviors*** | | **Total change** | **Increased** | **Decreased** |
| --- | --- | --- | --- | --- |
|  |  | **%** | **%** | **%** |
| *Contemplative practices (n = 27)* | Meditation (n = 25) | 92.6% | 92.6% | 0% |
|  | Mindfulness (n = 23) | 85.2% | 85.2% | 0% |
|  | Spiritual practices (n = 22) | 81.5% | 77.8% | 3.70% |
|  | Stress reduction practices (n = 18) | 66.7% | 66.7% | 0% |
|  | Prayer (n = 18) | 66.7% | 66.7% | 0% |
| *Diet and nutrition (n = 26)* | Consumption of sugar-based foods and drinks (n = 21) | 80.8% | 3.85% | 76.9% |
|  | Consumption of vegetables and fruits (n = 21) | 80.8% | 76.9% | 3.85% |
|  | Consumption of processed foods (n = 21) | 80.8% | 3.85% | 76.9% |
|  | Consumption of nuts and whole grains (n = 17) | 65.4% | 65.4% | 0% |
|  | Consumption of meat (n = 16) | 61.5% | 11.5% | 50% |
|  | Consumption of vegan/vegetarian meals (n = 14) | 53.8% | 46.2% | 7.69% |
|  | Consumption of legumes (n = 13) | 50% | 42.3% | 7.69% |
| *Time spent in nature (n = 26)* | Of “high quality” time spent in nature (n = 24) | 92.3% | 92.3% | 0% |
|  | In surrounding greenspace (n = 21) | 80.8% | 80.8% | 0% |
|  | In surrounding bluespace (n = 21) | 80.8% | 76.9% | 3.85% |
|  | In urban bluespace (n = 17) | 65.4% | 61.5% | 3.85% |
|  | Gardening (n = 16) | 61.5% | 61.5% | 0% |
|  | In urban greenspace (n = 15) | 57.7% | 53.9% | 3.85% |
| *Eating patterns (n = 25)* | Eating according to one’s body needs (n = 23) | 92% | 92% | 0% |
|  | Flexible eating (n = 23) | 92% | 80% | 12% |
|  | Slow, mindful eating (n = 19) | 76% | 76% | 0% |
|  | Binges and cravings (n = 18) | 72% | 4% | 68% |
|  | Enjoyment of a broader range of foods without guilt (n = 15) | 60% | 56% | 4% |
|  | Ability to balance calories in vs calories out (n = 11) | 44% | 44% | 0% |
|  | Purgative behaviors (n = 4) | 16% | 4% | 12% |
| *Physical activity (n = 24)* | Yoga (n = 21) | 87.5% | 83.3% | 4.17% |
|  | Dancing (n = 19) | 79.2% | 70.8% | 8.33% |
|  | Walking (n = 19) | 79.2% | 79.2% | 0% |
|  | Hiking (n = 14) | 58.3% | 54.2% | 4.17% |
|  | Physical exercise (n = 13) | 54.2% | 45.8% | 8.33% |
|  | Swimming (n = 11) | 45.8% | 33.3% | 12.5% |
|  | Cycling (n = 9) | 37.5% | 29.2% | 8.33% |
|  | Running (n = 9) | 37.5% | 25% | 12.5% |
|  | Martial arts (n = 7) | 29.2% | 16.7% | 12.5% |
|  | Pilates (n = 6) | 25% | 16.7% | 8.33% |
|  | Team sports (n = 6) | 25% | 4.17% | 20.8% |
| Personally meaningful social activities *(n = 24)* | Social gatherings (n = 21) | 29.2% | 29.2% | 0% |
|  | Community engagement (n = 16) | 66.7% | 66.7% | 0% |
|  | Joining a group (n = 14) | 58.3% | 50% | 8.33% |
|  | Volunteering (n = 7) | 29.2% | 29.2% | 0% |
|  | Religious services (n = 7) | 29.2% | 25% | 4.17% |
|  | Political participation (n = 5) | 20.8% | 12.5% | 8.33% |
| *Work-life balance (n = 19)* | Reducing time spent at work/working (n = 16) | 84.2% | 84.2% | 0% |
|  | Not working during leisure hours (n = 16) | 84.2% | 84.2% | 0% |
|  | Scheduling limits (n = 15) | 78.9% | 73.7% | 5.26% |

**Supplementary File 3 - Psychosocial mechanisms**

| Mechanism | Definition |
| --- | --- |
| Autonomy | Feeling an increased sense of personal ownership and wholehearted self-endorsement of reasons for making behavior change(s). |
| Awe | An experience of awe, wonder, and amazement, something vast that transcended their prior understanding of the world. |
| Bodily awareness | An increased sense of awareness toward one’s physical body, including physical symptoms related to their health. |
| Coherence / self-concordance | Feeling less fragmented and restless; more unified and at peace; a sense that their values and goals are in harmony. |
| Communitas | Feeling a greater sense of togetherness and shared humanity with other people. |
| Competence | Feeling a greater sense that life is a challenge to be taken on; an increased desire to seek out challenges, taking them on and exerting effort until personal growth is experienced. |
| Connection to nature | Feeling more connected to nature. |
| Emotional breakthrough | Overcoming challenging emotions or memories and thereby experiencing emotional release or breakthrough. |
| Health as an aspiration | A shift toward valuing “to be physically healthy” as an important life goal. |
| Health as an identity | A shift toward seeing being healthy as a defining aspect of themself or part of their “true self”. |
| Integrated emotion regulation | An increased capacity to openly and nonjudgmentally explore your emotions once they arise (vs. suppressing or failing to regulate your emotions. |
| Integration^*^ | An intentional integration process after the psychedelic experience at least partially supported by a facilitator, guide, or friend that was focused on health behavior change. |
| Intrinsic-extrinsic values | A change in values, such that the importance of self-acceptance, affiliation, and community increased, while the importance of financial success, appearance, and social recognition decreased. |
| Intuitive knowledge | Subjective judgment and knowledge that appears to be based on a gut feeling rather than learning specific. |
| Mortality acceptance | A shift toward accepting and coming to peace with one’s own mortality and eventual death. |
| Mystical experience | An intense experience characterized by a sense of oneness, sacredness, transcendence of time and space, and being beyond what words can fully describe. |
| Open-heartedness | An increased capacity to see the loveliness in others, no matter who they are or what they’ve done. |
| Perceived good-life coherence | Feeling a great coherence between their current lifestyle and their personal philosophy of what it means to live a good life. |
| Preparation^*^ | An intentional preparation for the psychedelic experience at least partially with a facilitator, guide, or friend that was focused on health behavior change. |
| Psychological flexibility | An increased ability to think and respond flexibly, especially in response to negative thoughts, feelings, and events. |
| Psychological insight | Gaining of a new perspective or clarity on one’s self or life (personality, behavioral patterns, emotions, or thoughts). |
| Quieted ego / ego dissolution | An experience of detached awareness of themselves as a part of (rather than distinct from) something much larger. |
| Relatedness | Feeling a greater sense of connection to other people, for extending warmth and regard toward others, and feeling it in return. |
| Self-efficacy | Personal beliefs about their abilities to change; for example feeling more confident in their skills and abilities when facing common obstacles. |
| Self-compassion | An increased sense of compassion or kindness for themselves, including their physical bodies. |
| Self-criticism | A decrease in the frequency and intensity of self-criticism that they engage in. |
| Sense of purpose in life | Feeling a greater sense of purpose in life. |
| Subjective / psychological well-being | Feeling more positive emotions, fewer negative emotions, and feeling more satisfied with their lives. |
| Values and committed action | A change in how much the person valued and was committed to making that health behavior change(s). |
